# Outcomes of the Health Data Research Network (HDRN) Canada and Canadian Longitudinal Study on Aging (CLSA) Partnership

**DOI:** 10.23889/ijpds.v9i5.2745

**Published:** 2024-09-18

**Authors:** Anne Hayes, Sophie Hogeveen, Jodi Gatley, Andrew Costa, Lauren Griffith, Aaron Jones, Sarah Youssef, Parminder Raina, Andrea Gruneir, Carrie Anne Whyte, Carla Hovey, Marie-Chantal Ethier, Donna Curtis Maillet, Mahmoud Azimaee, Erik Youngson, Tim Choi, Lindsey Gilbert, Magda Nunes De Melo, Lindsay Stewart, Kendra Lester, Katelyn Frizzell, Robyn Kydd, Mary-Ann Standing, Charles Burchill, Dave Towns, Jean-Francois Cantin

**Affiliations:** 1 Health Data Research Network Canada; 2 Canadian Longitudinal Study on Aging; 3 University of Alberta; 4 Canadian Institute for Health Information; 5 New Brunswick Institute for Research, Data and Training; 6 ICES; 7 Alberta Health Services; 8 Population Data BC; 9 Health Data Nova Scotia; 10 NL Health Services; 11 Secure Island Data Repository; 12 Manitoba Centre for Health Policy; 13 Institut de la statistique du Québec

## Objective and Approach

The Canadian Longitudinal Study on Aging (CLSA) is the largest national longitudinal cohort study, following ~50,000 adults across 10 provinces for at least 20 years. Health Data Research Network (HDRN) Canada is a distributed pan-Canadian network including members in all 13 provinces and territories. In February 2021, the organizations partnered with the objective to provide researchers with efficient access to multi-regional CLSA data linked to other health/health-related data through HDRN Canada’s data centres. A Steering Committee was convened including representatives from all parties to oversee the process from data sharing agreements (DSAs), negotiating terms of access, through to data transfer and linkage.

## Results

In Spring 2024, linkage was complete at four data centres (Ontario, New Brunswick, British Columbia, and Nova Scotia), with a DSA also in place for Newfoundland and Labrador. DSAs are nearing approval at three others (Manitoba, Prince Edward Island, and Quebec), and in progress in Alberta. Saskatchewan is working towards participation.

## Conclusions

The HDRN Canada-CLSA partnership is on the way to making linked provincial CLSA cohort data available in all participating jurisdictions, facilitated by streamlined agreements and processes.

## Implications

Collaborative efforts of organizations such as HDRN Canada and CLSA to foster data linkages and streamline data access at a multi-regional level offers an unparalleled opportunity to explore the intersection between aging and health care utilization. Research enabled by this initiative will inform health care decision making and evidence-based policy development at multiple levels, from the individual to multiple levels of government.

